# Incidence, identification and antibiotic resistance of *Salmonella *spp. in the well waters of Tadla Plain, Morocco

**DOI:** 10.1038/s41598-024-61917-3

**Published:** 2024-07-04

**Authors:** Fatima Zahra Hafiane, Latifa Tahri, Mohamed El Jarmouni, Ahmed M. Reyad, Mohammed Fekhaoui, Mohamed O. Mohamed, Ehab A. Abdelrahman, Samar H. Rizk, Gharieb S. El-Sayyad, Walid F. Elkhatib

**Affiliations:** 1 Geo-Biodiversity and Natural Patrimony Laboratory GEOPAC Research Center Scientific Institute, Mohammed V University in Rabat, Ibn Battuta Av, B. P1040, Rabat, Morocco; 2 Managem Group, Twin Center, Tour A, BP 5199, Casablanca, Morocco; 3 Botany and Microbiology Department, Faculty of Science, Beni-Suef University, Beni-Suef, 62511 Egypt; 4 Biotechnology and Genetic Engineering Department, Faculty of Agriculture, Ain Shams University, Cairo, Egypt; 5 Department of Chemistry, College of Science, Imam Mohammad Ibn Saud Islamic University (IMSIU), 11623 Riyadh, Saudi Arabia; 6 Chemistry Department, Faculty of Science, Benha University, Benha, 13518 Egypt; 7 Department of Biochemistry, Faculty of Pharmacy, Ahram Canadian University, Giza, Egypt; 8 Department of Biochemistry, Faculty of Pharmacy, Galala University, Suez, Egypt; 9 Department of Microbiology and Immunology, Faculty of Pharmacy, Ahram Canadian University, Giza, Egypt; 10 Microbiology and Immunology Department, Faculty of Pharmacy, Ain Shams University, African Union Organization St., Abbassia, Cairo, 11566 Egypt; 11 Department of Microbiology and Immunology, Faculty of Pharmacy, Galala University, Suez, Egypt

**Keywords:** Water, *Salmonella* spp., Antibiotics, Multidrug-resistant, Tadla plain, Ecological epidemiology, Microbial ecology, Microbiology, Pathogenesis

## Abstract

Concerns about challenges with water availability in the Tadla Plain region of Morocco have grown as a result of groundwater contamination brought on by human activity, climate change, and insufficient groundwater management. The objective of the study is to measure the number of resistant bacteria in the groundwater of Beni Moussa and Beni Aamir, as well as to evaluate the level of water pollution in this area. 200 samples were therefore gathered from 43 wells over the course of four seasonal campaigns in 2017 and 2018. Additionally, the samples were examined to determine whether *Salmonella* species were present and if they were resistant to the 16 antibiotics that were tested. *Salmonella *spp. have been identified in 31 isolated strains in total, accounting for 18.02% of all isolated strains. Data on antibiotic resistance show that 58.1% of *Salmonella *spp. strains are multidrug-resistant (MDR); 38.7% of *Salmonella* strains are tolerant to at least six antibiotics, 19.4% to at least nine antibiotics, 9.7% to four to seven antibiotics, 6.5% to at least eleven antibiotics, and the remaining 3.2% to up to twelve antibiotics. A considerable level of resistance to cefepime (61.29%), imipenem (54.84%), ceftazidime (45.16%), ofloxacin (70.97%), and ertapenem (74.19%) was found in the data. Consequently, it is important to monitor and regulate the growth of MDR in order to prevent the groundwater's quality from declining.

## Introduction

Groundwater quality problems are getting worse as a result of increased industry and population. Research has indicated that *Salmonella *spp. and other bacteria have polluted groundwater all around the world^1^. However, human activity, climate change, and insufficient groundwater management are to blame for the declining quality of groundwater, endangering human life^2–7^. Concerns over difficulties with groundwater quality have increased^8^, which makes these challenges environmental, social, and political issues worldwide^9^. In addition to the use of antimicrobial agents for human therapy, their use in animals to increase the production of food is unavoidable. Therefore, the investigation of the presence of antibiotic resistance in *Salmonella *spp. has become a necessity^10,11^.

*Salmonella*-associated infections in undeveloped countries are linked to an invasive disease that frequently results in septicemia with high rates of mortality^12^. The emergence of resistance in this bacterium against important antimicrobials such as fluoroquinolones^13,14^ and, more recently, extended-spectrum β-lactamases (ESBL) is particularly concerning^15^.

Most of the time, the strains of *Salmonella* cause gastroenteritis, which is typically mild and doesn't need to be treated, but can be fatal in newborns, young children, and those with weakened immune systems^16^.

According to research, pathogenic bacteria such as *Salmonella *spp., *Staphylococcus aureus*, *Listeria monocytogenes*, *Campylobacter *spp., and *Escherichia coli* O157:H7 are frequently identified in contaminated foodstuffs, particularly from animal products, which can be transmitted to the human body^17,18^.

Antimicrobial resistance (AMR) poses a serious threat to human health, making it more difficult to treat an increasing number of illnesses globally and driving up the cost of care. The most popular and extensively used antimicrobials that cause bacterial cell lysis are the β-lactam antibiotics, which inhibit the production of bacterial cell walls. According to their chemical structures, these antibiotics have been divided into several groups, such as β-lactamase inhibitors, carbapenems, cephalosporins, cephamycins, and penicillins^19^.

*Salmonella* resistance to a variety of antimicrobials has evolved since the early 1990s, and it represents a serious public health concern. *Salmonella* is widely distributed in the environment, and some specific host conditions render humans vulnerable to *Salmonella* infections. Antimicrobial resistance, prevalence, virulence, and adaptability are all on the rise, posing a global health threat. Antibiotics are used by livestock and poultry producers to treat a variety of illnesses and infections, as well as, growth boosters in low doses. These treatments support the animal’s health and well-being^9^.

However, antibiotics have also been connected to the emergence of genes that confer bacterial resistance, which lowers the effectiveness of antibiotics^20^. Multidrug-resistant bacteria are produced as a result of intensive antimicrobial usage which are prevalent in food and water^21,22^.

Agricultural, urban, and industrial activities have all contributed to the potential bacteriological contamination of groundwater supplies in recent decades. Antibiotics are commonly used in agriculture to prevent and treat disease while also promoting growth. As a result, animal feces may contain resistant bacteria, as well as antibiotic residues, causing selective pressure on microbes and affecting populations in the surrounding environment^23^.

Owing to the fact that illnesses brought on by ESBL-producing bacteria are resistant to several antibiotics, including third-generation cephalosporins, the rapid introduction and spread of ESBLs presents a serious risk to public health^24^. When manure is applied, pollutants like antimicrobial agents, resistant bacteria, and resistance genes are concentrated and mobilized in the soil. Eventually, through runoff, these pollutants reach the water^25^.

Antibiotic-resistant bacteria should be closely observed in water sources, as water serves as a conduit between the three primary reservoirs: soil, animals, and humans^17,26,27^.

In the selected study area, the groundwater is accessible through wells and boreholes. The combination of population growth in this region and its undeveloped economy has caused anarchic rural and urban expansion, with the multiplication and extension of agricultural yield and water demand; this leads to fundamental difficulties in drinking water supply. Therefore, the use of polluted groundwater has unfortunately become mandatory^28^.

To guarantee that all necessary steps are taken to ensure groundwater quality, this study describes and evaluates the antimicrobial resistance profile of *Salmonella* species collected from groundwater as well as the prevalence rate of these species.

## Materials and methods

### Study area

**Figure 1:**
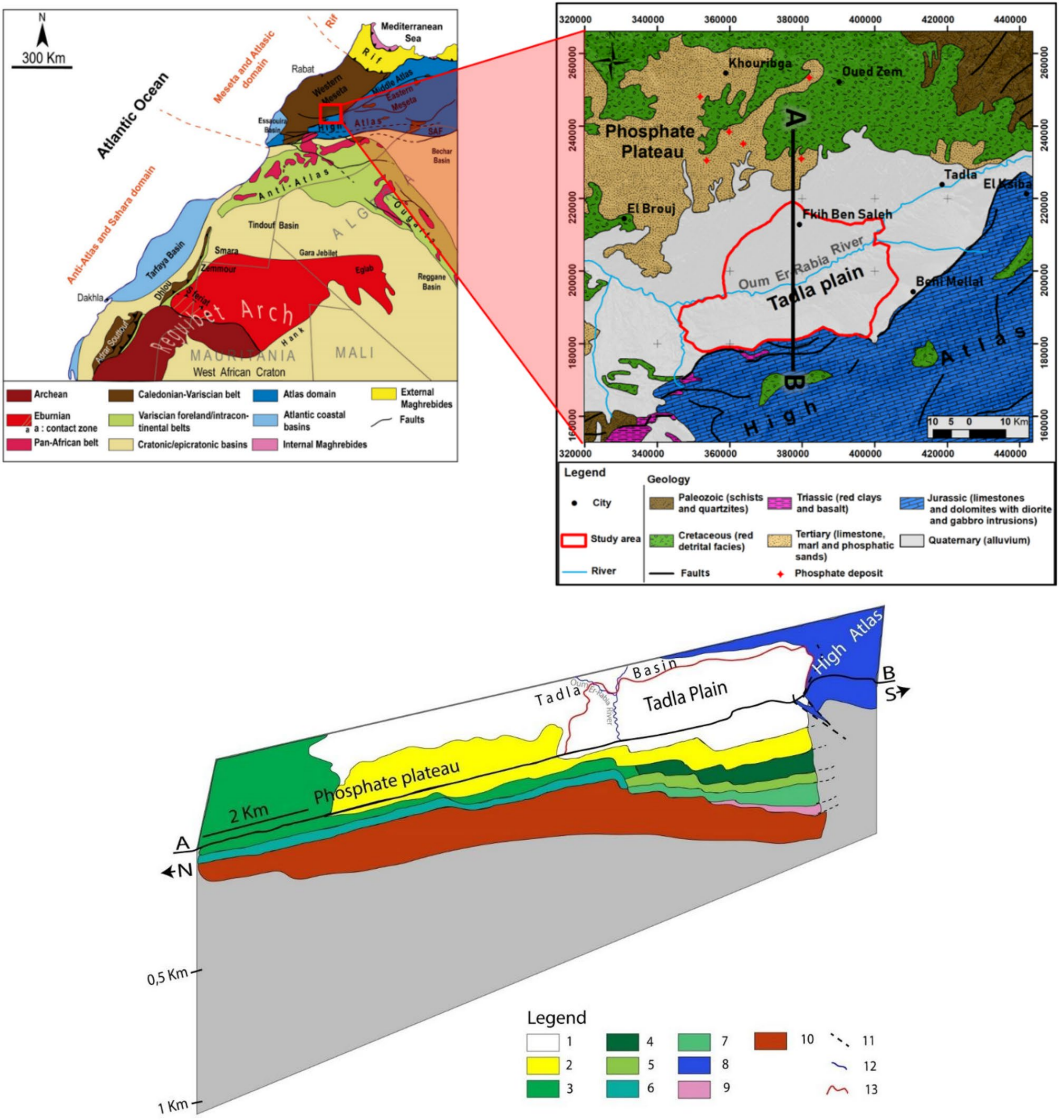
(**1-1**) Regional geological framework (modified from Michard et al.^65^ and El Kiram et al.^64^). (**1-2**) Tadla Basin A–B Geological Section/Tadla Plain Aquifer Overlay, modified from El Kiram et al.^64^.

The study occupies an area of approximately 10,000 km^2^ of Tadla Plain. This area is formed by the phosphate plateau in the north, the Tadla plain in the south, and the eastern Bahira in the west (Fig. 1-1). On the primary base of the Meseta lies a major unconformity of Cretaceous and Tertiary marine formations, which is largely extended to the south and gradually thickening under the detrital filling of the Mio-Plio-Quaternary of the Tadla plain.

**Figure 2:**
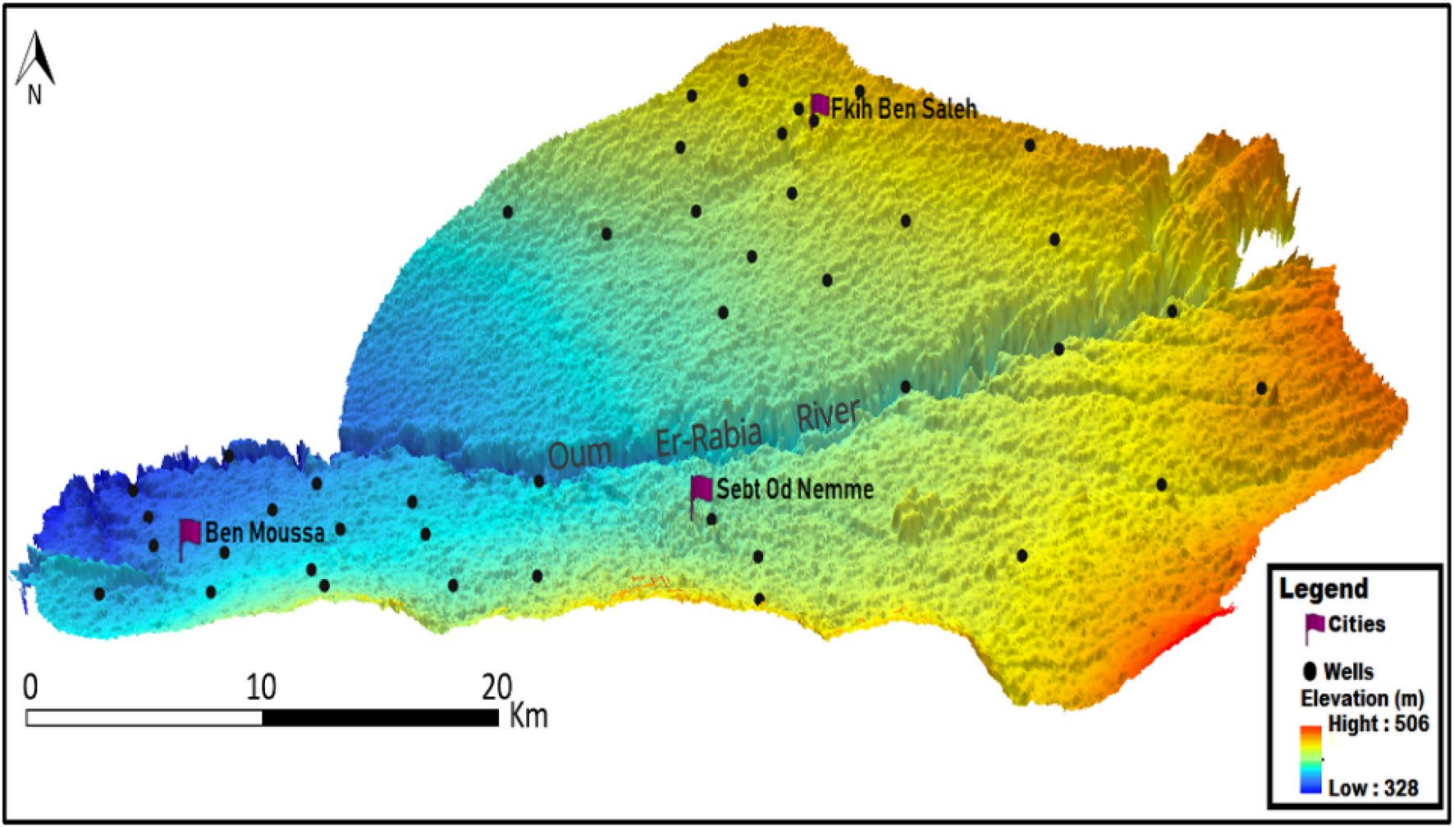
3D Model; emplacement of the 43 wells on both sides of Oum Er-Rbia River. The realization of this map was made by the Kriging method with the software Surfer 12 on a layer in terrain. This spatial modeling method allows from the points to have a representation on the entire surface of the area studied; the Beni Moussa and the Beni Aamir groundwater under a 3D model, in order to estimate the spatial location in a more accurate way.

In this plain, groundwater resources are made up of a multilayer aquifer system with groundwater, the confined aquifers of the Eocene and Turonian are estimated at 440 million m^3^, including 190 million m^3^ for the Beni Aamir and 250 million m^3^ for the Bni Moussa^29^. The Tadla aquifer system is a superposition of several aquifers (Fig. 1-2). Figure 1-2 is made by ArcGIS 10.5 software (including the ArcMap extension), from a shapefile that we got according to the agency of the hydraulic basin of the Oued Oum Rbiaa in Beni Mellal city.

The study covered 43 groundwater wells (Fig. 2) that were distributed as follows: 26 wells at the Beni Moussa aquifer (60.5% of the wells studied) and 17 wells at the Beni Aamir aquifer (39.5% of the wells studied). Water samples have been collected from the irrigated area during four campaigns. All samples are conserved at 4 °C in the laboratory until analysis time^30^.

### Sampling

A total of 387 water samples were collected during four sampling campaigns from 43 wells. Each of these wells is the subject of a four-sample bacteriological survey, following the seasonal variation.

Sterile polyethylene vials of 1 L were used for the collection of water samples. The vial is closed under the required aseptic conditions until the time of analysis. Before use, the vials are first washed and rinsed with distilled water. All analyses were carried out as quickly as possible within 24 h of sampling. After accomplishing the analyses, the samples were stored at a temperature of 4 °C.

### Detection of *Salmonella *spp.

*Salmonella *spp. generally exist in a precarious physiological state and in small numbers compared to the large and varied bacterial flora. To identify *Salmonella *spp., the process passes through three stages: pre-enrichment, enrichment, isolation, and identification; the comprehensive examination also includes the antibiogram. The set processing requires at least 96 h.

Detection of *Salmonella *spp. involves filtration of water samples (volume of 1000 mL) through the membrane filter, followed by a pre-enrichment of the membrane filter in buffered peptone water (oxoid: cm0509) of 100 mL per bottle, and then incubation at 37 °C for 20–24 h. Each bottle was subjected to a second enrichment on Rappaport Vassiliadis soya medium^31^; a broth with malachite green and magnesium chloride (svr; oxoid: cm0866) in tubes at a rate of 0.1 mL at 42 °C for 24 h, and isolation of *Salmonella *spp. was performed on Hektoen agar medium (oxoid: cm0419)^32^.

The hektoen agar plates were subjected to a second isolation on (Xylose-Lysine-Deoxycholate) xld agar (oxiod cm0469)^33^. After incubation of the petri dishes, *Salmonella* spp. will appear in the form of colonies of gray blue color with a black center. Morphological and biochemical identification were performed by seeding the isolated colonies in Kligler tubes (oxoid: cm0033) as previously described^34^.

### Serology of *Salmonella *spp.

Antigens including VI (envelope somatics), O (somatic), and H (flagellar) can be detected by slide agglutinations with the appropriate sera. In this study, O antigen was detected with anti O mixtures: OMA, OMB, and OMC. It was not available to test the anti-H sera corresponding to the group and thus to determine the name of the serovar following the absence of serums.

### Antibiotic susceptibility

The antibiogram was performed for each isolate by the Mueller Hinton agar diffusion method according to the recommendations of the European Committee on Antimicrobial Susceptibility Testing^35^. From a fresh bacterial culture on nutrient agar, a bacterial suspension was prepared in sterile normal saline to achieve a turbidity equivalent to 0.5 McFarland standard, which corresponds to an inoculum of about 1 to 2 × 10^8^ CFU/mL.

Swabs over the entire surface of the Mueller Hinton agar were spread in three directions with the bacterial suspension, after which the antibiotic discs were deposited. The agar plates were incubated for 18–20 h at 37 °C. After incubation, the diameters of inhibition zones were measured in mm to determine the antibiotic susceptibility profile for each isolate.

The antibiotics used in the current susceptibility study involved: Cephalosporins (Ceftazidime: CAZ; 30 μg, Cefotaxime: CTX; 30 μg, and Cefepime: CFP; 30 μg), Polymyxin (Colistin: CN; 10 μg), Aminoglycoside (Gentamicin: GN; 10 μg), Sulfonamides (Trimethoprim/sulfamethoxazole (Cotrimoxazole): SXT; 25 μg), Cycline (Tetracycline: TE; 30 μg), Macrolide (Erythromycin: E; 15 μg), Quinolones and fluoroquinolones: (Nalidixic acid: NA; 30 μg, Ofloxacin: OFX; 5 μg, and Norofloxacin NOR; 10 μg). Penicillins/β-Lactamase inhibitors: (Amoxicillin/clavulanic acid: AMC; 30 μg, and Ampicillin/Sulbactam: SAM; 30 μg). Carbapenems: (Ertapenem: ERT; 10 μg, and Imipenem: IPM; 10 μg) and Monobactams: (Aztreonam: ATM; 30 μg).

The efficiency of the *Salmonella* strains isolation has been verified according to CA-SFM/EUCAST, 2018 and MN 03.7.003, using a standard strain (*E. coli* ATCC 25922). The validity of the analytical methods was verified in the same way as in our previous study^27^.

## Results and discussion

### Spatio-temporal variation of *Salmonella *spp.

**Figure 3:**
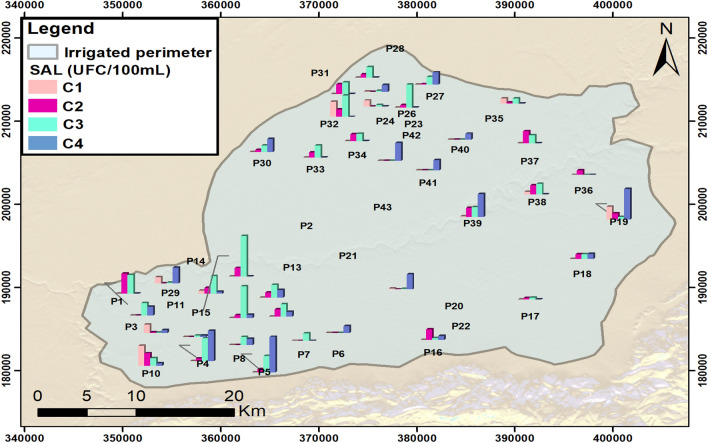
Geographical distribution of *Salmonella *spp. in the tested well water. C1, C2, C3, and C4 indicate 4 sampling campaigns. A total of 387 water samples were collected during 4 sampling campaigns from 43 wells. Each of these wells are the subject of 4 sample bacteriological survey, following the seasonal variation.

The results have shown that the well-analyzed water samples contain *Salmonella *spp. The presence of this microbe is noted in the well water during both seasons (dry and wet). The proportions of water samples that revealed the presence of *Salmonella *spp. with a prevalence of 57% in the wells were analyzed at the irrigated perimeter. Furthermore, *Salmonella *spp. could be detected in 387 water samples collected during two seasonal campaigns. The wells contaminated by such microbes are spread all over the wells of the two Bni Aamir (BA) and Bni Moussa (BM) aquifers (Fig. 3). The high prevalence of well contamination reveals values between a minimum of 1 CFU/100 mL (P11; P20; P25; P23; P29; P39) and a maximum of 80 CFU/100 mL (P2).

### Prevalence of resistant *Salmonella *spp. in the well water

The antibiograms of *Salmonella* isolates (n = 31) from the well water were determined. The antibiograms of *Salmonella *spp. revealed a high level of resistance.

**Figure 4:**
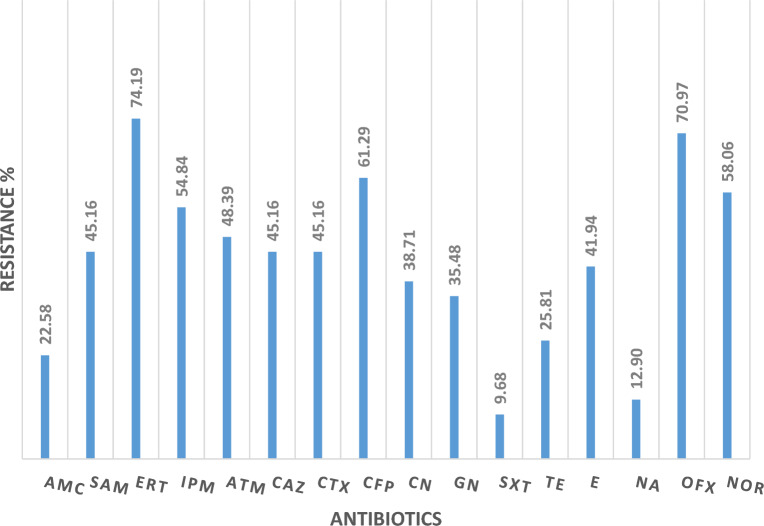
Percentage of antibiotic resistance of *Salmonella *spp. recovered from wells water. *AMC* Amoxicillin/Clavulanic acid, *SAM* Ampicillin + Sulbactam, *ERT* Ertapenem, *IPM* Imipenem, *ATM* Aztreonam, *CAZ* Ceftazidime, *CTX* Cefotaxime, *CFP* Cefepime, *CN* Colistin, *GN* Gentamycin, *SXT* Trimethoprime/sulfamethoxazole, *TE* Tetracycline, *E* Erythromycin, *NA* Nalidixic acid, *OFX* Ofloxacin, *NOR* Norofloxacin.

High levels of resistance were observed with different antibiotic classes, including carbapenems (74.19%; Ertapenem, and 54.84%; Imipenem), fluoroquinolones (70.97%; Ofloxacin and 58.06%; Norfloxacin), cephalosporins (61.29%; Cefepime and 45.16%; Ceftazidime and Cefotaxime), Monobactam (48.39%; Aztreonam), Penicillin/β-Lactamase inhibitors (45.16%; Ampicillin/Sulbactam), followed by macrolide (41.94%; Erythromycin) (Fig. 4).

### Prevalence of multidrug-resistant (MDR) *Salmonella *spp.

The MDR *Salmonella* strain is defined as a strain that is resistant to more than four antibiotics. The results (Table 1) revealed that 38.7% of the strains are resistant to at least 6 antibiotics, 19.4% of the strains are resistant to at least 9 antibiotics, 9.7% of the strains are resistant to at least (4, 5, and 7) antibiotics, 6.5% of the strains are resistant to at least 11 antibiotics, and finally 3.2% of the strains are resistant to at least 8 and 12 antibiotics.

**Table 1:** Classification of multi-resistant strains of *Salmonella* spp.

*Salmonella* isolates resistant to	Number of resistant isolates	% (MDR)
4 Antibiotics	3	9.7
5 Antibiotics	3	9.7
6 Antibiotics	12	38.7
7 Antibiotics	3	9.7
8 Antibiotics	1	3.2
9 Antibiotics	6	19.4
11 Antibiotics	2	6.5
12 Antibiotics	1	3.2
Total number	31	100

### *Salmonella *spp. resistance phenotype

Our results provide insight into both the analysis of resistance phenotypes of *Salmonella *spp. recovered from well water and categorizing these isolates according to Bush-Jacoby^36^.

**Figure 5:**
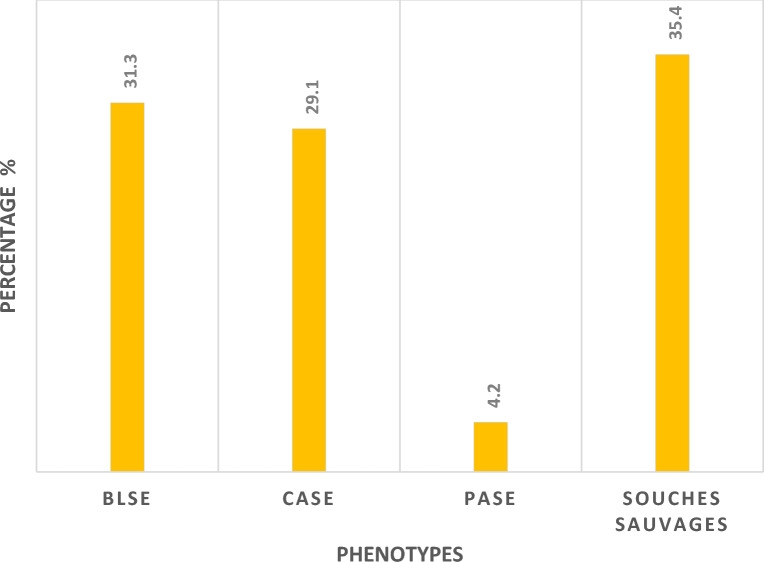
Resistance phenotypes of MDR *Salmonella* spp. recovered from water samples. Our results provide the analysis of resistance phenotypes. *BLSE* Extended-Spectrum Beta-Lactamase, *CASE* cephalosporinase, *PASE* Penicillinase, *Souches sauvages* wild phenotype.

These results show a significant preponderance of 10 extended spectrum B lactamases (31.3%). 9 salmonella strains had hyperproduction of cephalosporinase (29.1%), one strain had penicillinase production (4.2%), and 35.4% of the 11 strains had no acquired resistance, making them a wild phenotype (Fig. 5).

### β-Lactams resistance phenotypes

ESBL results in resistance to all penicillins and cephalosporins, in particular the third-generation cephalosporin C3G (CTX, CAZ) and monobactam (TMJ). On the other hand, the activity of imipenem has not undergone any change. The “champagne cork” synergy phenomenon results in the inhibition of enzymatic activity by clavulanic acid and is often detected between C3G and AMC^35^.

Penicillinase results in high-level resistance to SAM and is characterized by the inhibition of enzyme activity through clavulanic acid, which makes the AMC activity superior to the SAM activity, as shown in Fig. 5.

Cephalosporinase results in high-level resistance to first-generation cephalosporin C1G that shows decreased sensitivity to the cefalexin ofloxacin (FOX), as shown in Fig. 5. Furthermore, the activity of the second-generation cephalosporins C2G (Cephalosporinase Second Generation) and C3G (Cephalosporinase Third Generation) is slightly low; on the other hand, the activity of imipenem (IPM) remains normal.

Water can be considered the main link between the three main ecosystems: humans, animals, and soil, that circulates antibiotic resistance^37,38^ and moreover, antibiotic-resistant bacteria are transferred between these ecosystems through fecal discharges.

The presence of *Salmonella* in more than 90.6% of water samples during the four campaigns is non-compliant with Moroccan standards, which recommend that there be an absence of *Salmonella* in water samples. The existence of *Salmonella *spp. during all seasonal campaigns indicates that the contamination problem related to this microbe is an uninterrupted process. Human and animal excrements are the main source of *Salmonella* pollution because humans and animals eliminate it through the stool in cases of both illness and via asymptomatic carriers^39,40^.

Seasonal changes in environmental circumstances may affect the occurrence and persistence of *Salmonella*. For instance, *Salmonella* detection levels in the watersheds have been linked to both precipitation and temperature^41–43^. The hypothesis that environmental water plays a role in the transmission of human infections by acting either directly as a vector or indirectly as a reservoir for *Salmonella,* is supported by seasonal and spatial patterns in the prevalence of *Salmonella* in the aquatic environment, as well as the rate of *Salmonella* infection cases. However, there are many variables to take into account, mainly the complex interaction between *Salmonella*'s presence in aquatic water and the environmental conditions^41^.

The contaminated water is subsequently discharged into rivers, allowing antibiotics and antibiotic-resistant bacteria (AMRB) to spread downstream. Similarly, cattle effluents containing AMRB and antibiotics can contaminate waterways because contaminated pastures and fields might be directly connected to rivers and groundwater through run-off and infiltration. It has been reported^30,44,45^ that latrines, waste dumps, and sumps are located in the immediate environment (less than 15 m) around the wells. Under these conditions, the contamination of well water by discharged excrement is favored by storm water runoff and infiltration, according to previous studies^46,47^.

Carvalho et al.^48^ detected *Salmonella* in all samples of shrimp farming and freshwater environments in the Northeast region of Brazil; however, concentrations were poorly linked with bacterial indicators of fecal contamination. *Salmonella* may, therefore, belong to the autochthonous microbiome. Furthermore, contact with the droppings of domestic or wild animals at any stage of production or handling could spread microbial contamination^49–51^. In fact, waterborne transmission has been identified as the primary mode of transmission in both drinking water and waste water^24^. Interpersonal contact is typically a key mechanism of fecal–oral transfer^52^.

There is significant evidence of resistant gene exchanges between environmental bacteria and human pathogens, which can significantly occur in aquatic environments^53^.

Water, as previously mentioned, appears to play a significant role in the dissemination of antibiotics and AMRB to natural ecosystems. As an example of this role, studies on marine animals have revealed that they carry a wide range of AMRB^54^ and that AMRB prevalence has been steadily growing over the previous decade^55^. In the Oldman River watershed in Alberta and the Canada Salmon River watershed in southwestern British Columbia, analyses of resistant bacteria, such as *Salmonella *spp., suggested the possibility of contamination from human waste and a variety of domestic and wild animal species^56^. There have been prior reports of *Salmonella *spp. exhibiting multiple antibiotic resistance in animals, food, and water sources^12,57^.

In Tajikistan, major epidemics of multi-resistant strains disseminated by polluted water were documented in 1997. There were almost 6000 cases reported in Tajikistan's water-borne outbreak. Additionally, there was a concerning development of the epidemic strains having decreased susceptibility to ciprofloxacin^58^. The study of the antibiotic susceptibility of *Salmonella* suggested that the acquisition of resistance would be linked to the use of antibiotics in veterinary and human settings. This was justified by antibiotic resistance patterns and mechanisms affecting the majority of antibiotic classes. Resistance selectively reduced the activity of carbapenems and fluoroquinolones as well as cephalosporins^59^.

It has been reported that the *Salmonella *spp. isolates were most commonly resistant to streptomycin (12.2%), tetracycline (10.6%), sulfisoxazole (9.7%), and ampicillin (7.5%) in the Oldman River watershed in Alberta and the Canada Salmon River watershed in southwestern British Columbia^56^. These results are in agreement with those study, which showed 45.16% of resistance to Ampicillin/Sulbactam, 25.81% Tetracycline, and (9.68%) Trimethoprim/sulfamethoxazole.

This situation would be linked to the anarchic introduction of these molecules in human medicine with irrational antibiotic use (indication, dosage, or duration), which preceded the agri-food sector and particularly veterinary medicine in our country. In the current study, *Salmonella* spp. revealed a high level of resistance to Trimethoprim/sulfamethoxazole (90.32%), Nalidixic acid (87.1%), and Amoxicillin/clavulanic acid (77.42%).

Nine antimicrobials could be used to treat 19.4% of the evaluated *Salmonella* isolates, including six isolates that were multidrug resistant. The majority of *Salmonella* isolates had a 38.7% resistance to at least six antimicrobials, including 12 isolates with multidrug resistance.

The most widely used antibiotic in animal health, colistin, had an average sensitivity of 61.29% against the isolates of *Salmonella* found in this investigation. It was discovered that the susceptibility of *Salmonella* isolates to colistin was 100% sensitive and effective; nevertheless, previous research has demonstrated varied degrees of sensitivity^60,61^. However, *Salmonella* found in North India was totally resistant to colistin^62,63^. By participating in aquatic activities, the general population could be exposed to a health risk and a source of AMR transmission.

## Conclusion

In the research region, there is a significant danger to human and animal health due to groundwater pollution in the irrigated periphery of the Tadla Plain polluted by excrement, a source of several infections. According to our findings, 57% of the water samples from the well under examination contained *Salmonella *spp. But the main focus of our research is on the patterns of antibiotic resistance that *Salmonella *spp. exhibits. As a result, *Salmonella *spp. isolates' resistance phenotype and percentage of resistance to various antibiotic classes elevate the risk warning in this tested water.

## Data Availability

The datasets used and analyzed during the current study are available from the corresponding author on reasonable request**.**
